# Glial changes as result of cerebral amyloid angiopathy progression

**DOI:** 10.1002/alz.093053

**Published:** 2025-01-03

**Authors:** Katelynn E. Krick, Sherika N. Johnson, Colin B Rogers, Tiffany L. Sudduth, Erica M. Weekman, Donna M. Wilcock

**Affiliations:** ^1^ Indiana University School of Medicine/Stark Neurosciences Research Institute, Indianapolis, IN USA; ^2^ University of Kentucky College of Medicine, Sanders‐Brown Center on Aging, Lexington, KY USA; ^3^ University of Kentucky / Sanders‐Brown Center on Aging, Lexington, KY USA; ^4^ Indiana University School of Medicine, Stark Neurosciences Research Institute, Department of Neurology, Indianapolis, IN USA

## Abstract

**Background:**

Cerebral Amyloid Angiopathy (CAA) occurs at the intersection of Alzheimer’s disease and vascular contributions to cognitive impairment and dementia (VCID). In the human brain it occurs when amyloid beta (Aβ) aggregates in small/medium‐sized cerebral blood vessels, which contribute to hypoperfusion and cognitive decline by altering vascular function and integrity. The current study seeks to track the progression of CAA and associated neuroinflammation and glial cell changes in Tg2576 mice.

**Method:**

Tg2576 mice were aged to 8‐, 14‐, 20‐, and 27‐months and assessed for CAA pathology via histology. Gene expression was evaluated in hippocampal tissue by qPCR and posterior cortex by nanostring (ncounter Mouse Neuroinflammation and Mouse CVD Pathophysiology panels; in progress) to compare 8‐ and 20‐month APP and wildtype groups. Protein expression was evaluated via digital spatial profiling in the same APP mice, grouped by age or CAA presence compared to wildtype controls. CAA presence was defined as Aβ surrounding lectin‐positive vessels. Regions of interest were categorized as either positive or negative for CAA based on this criterion.

**Result:**

Congophillic plaque deposition along the vasculature increased in width, length, and area in a time dependent manner in both the frontal cortex and hippocampus. Astrocyte marker GFAP and proinflammatory receptor TNFR1 gene expression both increased at 20 months compared to 8 months in the APP group. Of the protein profile assessed (Mouse Neural Cell Profiling and Mouse Glial Cell Subtyping), the most consistent changes were found in astrocyte markers. Both Aldh1l1 and S100B were increased at 20 months compared to 8 months of age in both APP and WT mice. GFAP protein expression was found to increase with both age and CAA.

**Conclusion:**

This study showed increased vascular amyloid deposition and astrocyte gene and protein expression over time, further supporting a role for astrocytes in etiology and/or reaction to CAA.

